# Transitioning From Co-Location to Collaboration in Integrative Medicine: A Qualitative Thematic Analysis of Focus Group Discussions Aligned With the WHO Traditional Medicine Strategy (2014-2023)

**DOI:** 10.7759/cureus.107707

**Published:** 2026-04-25

**Authors:** Kawal Krishan, Danish Javed, Rachna Parashar, Monika Saini, Sana Anwar

**Affiliations:** 1 Department of Hospital Administration, All India Institute of Medical Sciences, Bhopal, Bhopal, IND; 2 Department of AYUSH, All India Institute of Medical Sciences, Bhopal, Bhopal, IND; 3 Department of Physiology, All India Institute of Medical Sciences, Bhopal, Bhopal, IND; 4 Department of Social Sciences, National Institute of Health and Family Welfare, New Delhi, IND; 5 Department of Community and Family Medicine, All India Institute of Medical Sciences, Bhopal, Bhopal, IND

**Keywords:** ayurvedic medicine, complementary therapies, focus groups, health care surveys, integrative medicine, qualitative research

## Abstract

Overview: In India, Integrative medicine combines conventional allopathic treatments with well-researched evidence-based complementary therapies and holds the promise of enhanced patient care. However, transitioning from mere co-location of these diverse medical systems to genuine collaboration poses significant challenges. This study explores the experiences and insights of medical professionals involved in integrative health clinics.

Method: A focus group discussion (FGD) was conducted with 23 participants, including medical officers and research officers from various traditional medicine systems, and allopathic faculty members from several All India Institutes of Medical Sciences (AIIMS) with established integrative health clinics. Moderated by representatives from the AYUSH Ministry and the allopathic medical faculty from the National Institute of Health and Family Welfare (NIHFW), the discussions provided a balanced view of integrative health practices.

Result: The thematic analysis of focus group discussions identified one overarching theme, “Integration in the Medical Field,” encompassing five major sub-themes: terminology and clarity, evidence-based practice, practical integration, challenges to integration, and policy and funding. Participants emphasized the need for clear definitions, robust evidence to support integrative practices, and improved interprofessional collaboration. Key challenges included communication gaps, resistance due to differing medical philosophies, and ethical and regulatory concerns. The findings also highlighted the importance of education, standardization, and supportive policy frameworks to facilitate effective and sustainable integration.

Conclusion: This study offers valuable insights into the current state of integrative medicine and presents practical recommendations for fostering effective collaboration between traditional and modern medical systems. Addressing educational, operational, research, and policy needs can help to move towards a truly collaborative model of integrative medicine that enhances patient care and supports healthcare providers.

## Introduction

The Indian system of medicine does not only comprise traditional Ayurveda, Unani, or Yoga, but also includes a vast field of ancient and family medicine traditions. Traditional medicine is an important healthcare resource in many developing countries throughout the world [1]. Despite its significance in meeting the global healthcare needs, the role of traditional medicine is often underestimated in many countries [2]. The unique traditional system of medicine practiced in India has been majorly categorized into five distinctive categories: Ayurveda, Yoga and Naturopathy, Unani, Siddha, and Homoeopathy (AYUSH). Modern medical science, despite so many achievements and progress, is not always accessible to every person, posing a big challenge to attain the objective of universal health coverage [3]. Acknowledging the contribution of traditional medicine to health, wellness, people-centered healthcare, and universal health coverage, WHO seeks to bring traditional medicine into the mainstream of healthcare, appropriately, effectively, and above all, safely [4]. The WHO Traditional Medicine Strategy: 2014-2023, developed in response to the World Health Assembly resolution on traditional medicine, aims to support member states in developing proactive policies and implementing action plans that will strengthen the role that TM plays in keeping populations healthy [5].

The healthcare landscape is increasingly recognizing the value of integrative medicine (IM), which combines conventional allopathic treatments with traditional and complementary medicine systems such as AYUSH [6]. This approach aims to enhance patient care by leveraging the strengths of various medical traditions. However, transitioning from mere co-location of different medical systems to genuine collaboration presents numerous challenges and opportunities [7].

Integrative medicine seeks to provide a holistic approach to health and well-being, addressing the physical, mental, and spiritual aspects of patients [8]. Despite the potential benefits, the integration of diverse medical practices into mainstream healthcare systems has been fraught with obstacles [9]. These include differing medical philosophies, varying levels of acceptance among healthcare providers, regulatory issues, and the need for evidence-based practices [10,11].

This study aims to explore the experiences, challenges, and recommendations of medical professionals involved in integrative health clinics. By understanding these perspectives, we can identify strategies to facilitate more effective collaboration and improve patient outcomes.

To achieve this, we conducted focus group discussions (FGDs) with a diverse group of participants, including medical officers and research officers from AYUSH streams, as well as allopathic faculty members from various All India Institutes of Medical Sciences (AIIMS), and research organizations like the Indian Council of Medical Research (ICMR). These institutions have established integrative health clinics, providing a unique setting for examining how different medical systems can work together in clinical practice. This FGD was a part of a one-week training course titled “Building Knowledge Base and Awareness about Traditional and Complementary Medicine” conducted at the National Institute of Health and Family Welfare (NIHFW), New Delhi, from 26th February to 1st March 2024, and the FGD was conducted at the end. This training imparted here was an initiative taken by NIHFW to find out the possibilities and challenges for IM in India.

The FGDs were moderated by representatives from both the AYUSH Ministry and the allopathic medical faculty from NIHFW, ensuring a balanced discussion that reflects the realities and aspirations of both traditional and modern medical practitioners. This qualitative analysis provided rich insights into the current state of integrative medicine, highlighting the successes, barriers, and future directions for fostering genuine collaboration in clinical settings. Focus group discussion (FGD) was chosen as it facilitates dynamic interaction, allowing participants to build upon each other's experiences, thereby generating deeper insights into complex interdisciplinary challenges.

Despite increasing policy-level emphasis on integrative medicine, there remains a significant gap in understanding how healthcare professionals perceive and operationalize integration in real-world clinical settings. Existing literature predominantly focuses on theoretical frameworks or quantitative outcomes, with limited qualitative exploration of practitioner experiences, especially in the Indian context. The primary objective of this study was to explore and analyze the perceptions, experiences, and challenges faced by healthcare professionals regarding the integration of AYUSH systems with modern medicine in clinical settings.

Secondary objectives included: (1) identifying key thematic domains influencing integrative practices, (2) understanding operational, educational, and policy-level barriers, and (3) generating actionable recommendations to facilitate the transition from co-location to collaborative integrative healthcare models.

## Materials and methods

Study design

This study employs a qualitative research design using a focus group discussion (FGD) to explore perspectives on integrating traditional and complementary medicine systems (AYUSH) with allopathic medicine. The FGD approach allows for in-depth discussions, providing rich data on participants' experiences, opinions, and suggestions regarding integrative health practices.

Inclusion Criteria

Medical professionals (AYUSH and allopathic) participating in the NIHFW training program, individuals with at least two years of clinical/research experience, and those willing to participate and provide informed consent were included. 

Exclusion Criteria

Participants unwilling to be recorded and with no prior exposure to integrative medicine practices were excluded.

Participants

The FGD included a diverse group of approximately 23 participants, comprising medical officers and research officers from various traditional medicine systems (AYUSH). Other participants included faculty members from the allopathy branches of various All India Institutes of Medical Sciences (AIIMS) and ICMR institutes, who are mainly involved in clinical and research activities, including integrative medicine.

This diverse participant base ensures comprehensive insights from various medical disciplines involved in integrative healthcare. Their characteristics are shown in Table 1.

**Table 1:** Showing Participants Characteristics * The term “faculty” refers specifically to the official designation assigned to teaching-designated academic staff (such as assistant professors, associate professors, and professors) involved in clinical, teaching, and research activities.

Parameters	Values
Age; mean ± SD (years)	43.63±11.76
Gender	
Male	11
Female	12
Field	
Allopathy	6
Ayurveda	8
Unani	4
Homeopathy	1
Siddha	1
Basic science	2
Nursing	1
Designations	
Faculty*	6
Medical officers	5
Research officers	10
Scientist	2
Experience; mean ± SD (years)	14.21±8.97

Moderator and facilitator roles

Two moderators facilitated the discussions: a moderator from the AYUSH Ministry provided expertise in the traditional and complementary medicine system, and a moderator from the Allopathic Department of Health and Family Medicine ensured balanced perspectives and relevance to modern medical practices.

The session was conducted by a faculty member from the NIHFW, Delhi, ensuring smooth facilitation and adherence to the discussion guidelines.

Venue and setting

The FGD was held at a conference hall of NIHFW, Delhi, a neutral and conducive environment for open and unbiased discussion. The setting allowed for a comfortable and confidential atmosphere, encouraging participants to share their genuine thoughts and experiences.

Procedure

Preparation

Participants were informed about the study's purpose and the importance of their contributions. Consent was obtained from all participants. A semi-structured discussion guide was developed, covering key topics related to integrative health care.

Discussion Guide

The guide included questions on the integration of traditional and modern medicine, challenges faced, potential benefits, successful case studies or examples, and recommendations for policy and practice improvements.

Conducting the FGD

The session began with an introduction by the NIHFW faculty, explaining the study's objectives and the flow of the discussion. Moderators ensured that each participant had an opportunity to speak and that the discussion remained focused on the key topics. Probing questions were used to elicit deeper insights and clarify responses.

Recording and Transcription

The session was audio-recorded to ensure accurate data capture. Notes were taken by an assistant to supplement the recordings. The audio recordings were transcribed with the help of the Transkriptor app into verbatim for analysis (free to use).

Data analysis

Thematic analysis was conducted following Braun and Clarke’s six-step framework. Initial open coding was performed independently by two researchers. Codes were iteratively refined and grouped into categories, sub-themes, and overarching themes using a trial version of the NVivo 15 software (Lumivero, Burlington, MA). Inter-coder agreement was ensured through discussion and consensus.

Transcripts were reviewed for accuracy against the audio recordings. Any discrepancies were resolved by re-listening to the recordings and were coded using thematic analysis. Codes were developed both deductively (based on the discussion guide) and inductively (emerging from the data). To minimize bias, purposive sampling ensured representation from multiple disciplines. Dual moderation (AYUSH and allopathic experts) ensured balanced perspectives. Independent coding by two researchers was performed, followed by consensus discussion to reduce interpretive bias.

 Coded data were organized into themes and sub-themes. Themes were reviewed and refined to ensure they accurately reflected the participants' perspectives. Findings were triangulated with notes taken during the session and any relevant literature to enhance the validity of the results. All participants provided informed consent prior to participation. Participants' identities were kept confidential, and data were anonymized during transcription and analysis. The study protocol was reviewed and approved by the relevant ethics committee at NIHFW and received approval via NIHFW/Academic/IEC-HR/Pub/2024-1.

The FGD represents the views of a specific group of medical professionals and may not be generalizable to all healthcare settings. The presence of moderators from both AYUSH and allopathic backgrounds could introduce bias; however, their expertise was essential for balanced discussions. This FGD provided valuable insights into the integration of traditional and complementary medicine with allopathic practices. The methodology ensured a comprehensive understanding of the participants' perspectives, contributing to the development of effective integrative health care strategies.

## Results

**Figure 1:**
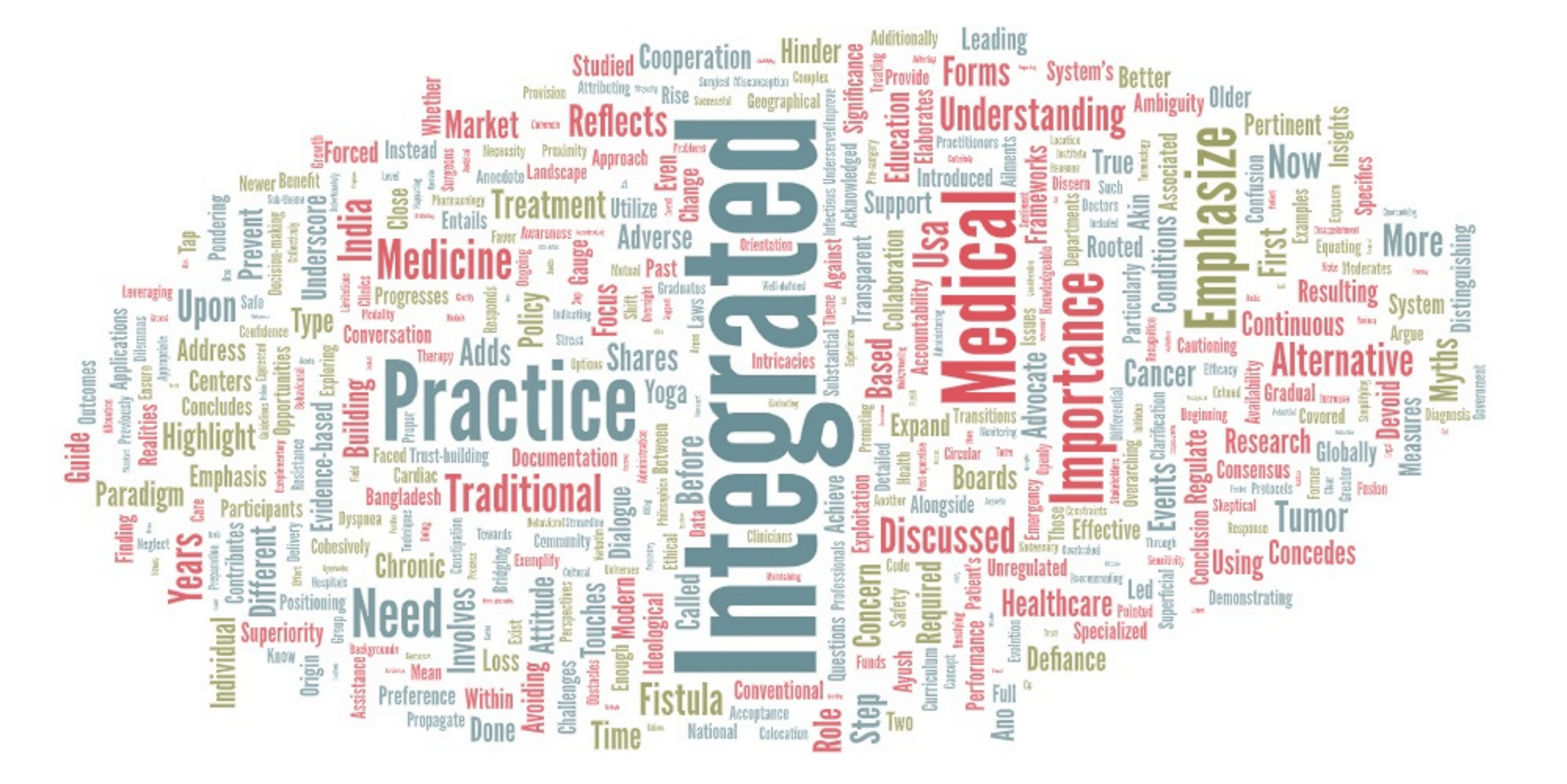
Word cloud showing the frequency of words spoken by the participants Image created by Dr. Danish Javed using NVivo 15 software (Lumivero, Burlington, MA).

The frequency of words spoken by the participants has been represented as a word cloud (Figure 1). The analysis of Focus Group Discussions (FGDs) yielded one overarching theme--“Integration in the Medical Field”--which encompassed five major sub-themes: Terminology and Clarity, Evidence-Based Practice, Practical Integration, Challenges to Integration, and Policy and Funding. Within these sub-themes, a total of 15 codes and 29 categories were identified, reflecting a comprehensive understanding of the multifaceted process of integrating various medical systems (Table 2).

**Table 2:** Result showing various theme, sub-themes, codes and category of FGDs Theme: Integration in the Medical Field

Sub-theme	Code	Category
Terminology and Clarity	Importance of Clear Terminology	(a) Emphasizing the necessity of distinguishing between integrative, complementary, alternative, and traditional medicines. (b) Avoiding confusion and ambiguity, especially around the term "alternative medicine." (c) Participants emphasized the ambiguity in terminology: ‘There is confusion between integrative and alternative medicine, which affects implementation at ground level.’ (Participant 3)
	Cultural and Medical Context	(a) Defining conventional, traditional, alternative, and complementary medicines within cultural and medical contexts. (b) Examples illustrating each type of medicine in practice.
	Coexistence and Complementarity	Focusing on the coexistence and complementarity of different medical approaches alongside standard care.
Evidence-Based Practice	Emphasis on Evidence	(a) Highlighting the necessity for integration to be rooted in evidence to ensure safety and efficacy. (b) Discussing examples like the fusion of yoga with cardiac intervention to illustrate evidence-based integration. “Without strong evidence, integration will not be accepted by mainstream clinicians.” (Participant 7)
	Legal and Regulatory Considerations	(a) Addressing legal implications and the importance of adherence to regulations in integrative practices. (b) Discussing the need for cross-referencing among different medical approaches to ensure legal compliance.
	Research and Documentation	(a) Stressing the importance of standardization, research, and documentation to validate the effectiveness of integrated practices. (b) Citing the need for continuous monitoring and regulation to prevent unethical practices.
Practical Integration	Operational Integration	(a) Discussing practical aspects like operational integration in emergency care and public health initiatives. (b) Examples of successful integration models in hospitals.
	Mutual Understanding and Collaboration	(a) Emphasizing the importance of mutual acceptance and collaboration among healthcare professionals. (b) Highlighting the need for sensitization and understanding each system's strengths and limitations.
	Education and Training	(a) Advocating for the inclusion of basic exposure to alternative treatments in medical education. (b) Discussing the gradual increase in acceptance and understanding of alternative practices over time.
Challenges to Integration	Communication Gaps	(a) Addressing challenges like communication gaps and differing educational backgrounds among medical professionals. (b) Discussing the need for improved communication to facilitate effective integration. “Communication gaps between systems are the biggest barrier to collaboration.” (Participant 12)
	Resistance and Attitudinal Shifts	(a) Reflecting on past resistance to alternative medicine and the need for attitudinal shifts towards acceptance. (b) Emphasizing the continuous effort required to build confidence within the medical community.
	Ethical and Accountability Concerns	(a) Highlighting concerns about accountability in cases of adverse events resulting from integrated treatments. (b) Stressing the importance of differential diagnosis and evidence-based decision-making in such scenarios.
Policy and Funding	Policy Frameworks	(a) Discussing the lack of proper guidelines and policy frameworks for integration. (b) Calling for more concerted efforts at the government level to address this gap.
	Funding Allocation	(a) Highlighting concerns about the allocation of funding from AYUSH to various medical institutions. (b) Emphasizing the need for performance-based funding and addressing disparities in funding allocation.
	Collaboration for Improvement	(a) Stressing the need for collaboration to address regulatory constraints and improve patient care.(b) Emphasizing the importance of behavioural change at both individual and systemic levels for better integration.

The sub-theme "Terminology and Clarity" highlighted the participants’ emphasis on the need for precise definitions and differentiation among integrative, complementary, alternative, and traditional systems of medicine. Participants underscored the importance of avoiding ambiguity, particularly surrounding the term "alternative medicine," and contextualizing these concepts within both cultural and medical frameworks. Examples from practice illustrated how diverse systems coexist and complement conventional medicine.

Under the sub-theme "Evidence-Based Practice," participants expressed that integration must be grounded in robust scientific evidence to ensure safety and efficacy. Discussions emphasized legal and regulatory compliance, the necessity of standardization, and the importance of ongoing research and documentation. Illustrative examples, such as the integration of yoga with cardiac care, were used to demonstrate how evidence-based practices can enhance outcomes.

The sub-theme "Practical Integration" focused on operational and collaborative aspects, including the successful inclusion of integrative models in hospital and public health settings. Participants stressed mutual respect, inter-professional understanding, and collaboration among practitioners of different systems. Additionally, they advocated for the inclusion of exposure to alternative therapies in medical curricula to foster greater acceptance and informed application.

The sub-theme "Challenges to Integration" revealed persistent issues such as communication barriers, differences in educational backgrounds, and resistance within the medical community. Participants noted that attitudinal shifts and continuous dialogue are essential to overcome skepticism and build trust. Ethical concerns-particularly regarding accountability and adverse outcomes-were also highlighted, with calls for evidence-based and differential diagnostic approaches to ensure patient safety.

Finally, the sub-theme "Policy and Funding" addressed systemic challenges, including the lack of clear policy frameworks and inequitable allocation of AYUSH funding across institutions. Participants advocated for performance-based funding models and stronger government initiatives to promote effective integration. Collaboration and behavioural change-both individual and institutional-were identified as crucial to achieving sustainable and ethical integration within the healthcare system.

## Discussion

The integration of alternative and complementary medicine (CAM) with conventional medical practices, i.e., integrative medicine, is an evolving field [12]. This study explores the transition from mere co-location of different medical practices to genuine collaboration within clinical settings. It draws upon qualitative data derived from focus group discussions, highlighting key themes, sub-themes, and specific codes that capture the essence of participants' experiences and insights.

Clarity in terminology and understanding

A significant theme that emerged is the necessity for clarity in terminology and understanding of integrative medicine. Participants emphasized the importance of basic exposure to alternative treatments during medical education. This foundational knowledge is crucial for fostering an environment where different medical systems can be understood and appreciated.

Education and research play a pivotal role in dispelling common myths about alternative treatments [13]. Participants highlighted the need for standardization, research, and documentation to ensure the safety and efficacy of integrative practices. This aligns with the call for evidence-based research, which is fundamental for validating integrative medicine practices and gaining broader acceptance within the medical community [14].

Coexistence and co-location

The theme of coexistence and co-location focuses on the practical aspects of integrating different medical practices within a single clinical setting. Participants identified communication gaps as a major barrier. There is a strong need for transparent communication and evidence-based decision-making to bridge these gaps. This includes fostering mutual understanding, education, and research to overcome the ideological superiority that often exists between different medical disciplines.

Building trust is also critical. Participants pointed out that regulation is necessary to prevent exploitation and ensure that integrative practices are conducted ethically. Moreover, trust-building involves addressing varying levels of skepticism and differing perceptions among practitioners towards integrative medicine [15].

Operational integration

Operational integration focuses on the practical steps needed to integrate different medical systems effectively. Participants noted the cultural significance of various medical practices, highlighting the importance of respecting and incorporating cultural beliefs and practices into integrative medicine.

Collaborative decision-making is essential, particularly in specialized areas such as tumor boards, where the integration of different medical perspectives can lead to better patient outcomes [16]. The necessity of integrating different medical systems, such as incorporating practices like the use of Kshar sutra in Fistula in Ano treatments, demonstrates the potential benefits of a holistic approach [17].

The focus group discussions underscored the importance of responsible integration practices. This includes ensuring that integrative medicine is practiced in a way that respects both traditional and modern medical knowledge, avoiding the pitfalls of unregulated or unethical practices [18].

Legal implications

The legal implications of integrative medicine are complex and multifaceted. Participants discussed the importance of cross-referencing and blending practices within the boundaries of established medical practice. This is crucial for maintaining accountability in cases of adverse events resulting from integrated treatments.

The discussions also highlighted the challenges posed by an unregulated market and the unethical practices that can arise in response to increasing trust in traditional medicine [19]. Implementing safety measures and establishing clear regulations are necessary to protect patients and ensure the credibility of integrative medicine [20].

Identifying areas for integration

Participants identified various countries, such as China, South Korea, Bangladesh, and India, as examples where integrative medicine has been successfully implemented. These examples provide practical insights into how mutual acceptance and collaboration can be achieved.

Understanding the practical aspects of integration is essential. This includes identifying areas where different medical systems can complement each other and developing strategies for effective collaboration. The focus group discussions emphasized the need for mutual acceptance and collaboration to advance integrative medicine [21].

The findings align with the WHO Traditional Medicine Strategy (2014-2023) [5], which emphasizes integration through regulation, research, and education. This study provides ground-level insights supporting these strategic pillars.

Strengths

This study provides real-world insights from multidisciplinary experts, applies systematic thematic analysis, and contributes to the limited qualitative literature on integrative medicine in India.

Limitations

This study has certain limitations. First, it is based on a single FGD, which may limit generalizability. Second, purposive sampling may introduce selection bias. Third, the absence of longitudinal data limits the assessment of implementation outcomes. However, the study provides rich contextual insights into integrative practices in India.

The complexities and challenges associated with integrative medicine require careful consideration. Participants stressed the importance of allocating funding based on performance rather than preference. This ensures that integrative medicine initiatives are supported by evidence of their effectiveness.

Achieving behavioral change at both individual and systemic levels is another significant challenge. Participants highlighted the need for ongoing education and awareness to promote integrative medicine and ensure its successful implementation in clinical settings [22].

Recommendations

Based on the qualitative analysis, several recommendations can be made to advance integrative medicine in clinical settings:

Educational Reforms

Integrating basic exposure to alternative treatments in medical education is essential. This could involve incorporating chapters on traditional medicine in various medical subjects and offering electives on integrative health practices during internships. For instance, including chapters on humors in human physiology or medicinal plants in pharmacology, concepts of Illness and Wellness in Pathology and Preventive and Social Medicine (PSM) can provide a foundational understanding of CAM. Introduction of practical elements such as Panchkarma in medicine, surgical and parasurgical techniques, yoga in psychiatry, and homoeopathy in dermatology can open new doors. AYUSH electives during MBBS internships and a three-year post-graduate course on integrative medicine for graduates from MBBS, AYUSH, and BDS on the lines of MD courses in family medicine may be initiated. Lowering entry barriers for AYUSH professionals in courses such as a master's in public health (MPH) degree and community medicine, and inclusion of integrative health practices in these courses, may be a good initiative.

Operational Strategies

Establishing patient education cells and integrative health counselling centres can facilitate better communication and understanding between practitioners and patients [23]. Training paramedical staff and higher management on integrative healthcare delivery can enhance operational efficiency and effectiveness. Promoting integrative health communication through meetings, seminars, conferences, and the constitution of integrated health clubs are welcoming steps [24]. Developing practice guidelines for different systems of medicine and formulating research guidelines can be a good step. Utilizing existing yoga protocols by Morarji Desai National Institute of Yoga (MDNIY) and creating guidelines for other AYUSH systems should be promoted. Focusing on 38 common ailments, defining cases, and correlating biomedical and traditional disease presentations, promoting health through home remedies, single herbs, and minor procedures, and providing ayurvedic management at various levels, from primary to tertiary care, ensuring continuum of care and proper referral systems can be operational strategies. Developing a dedicated portal for integrative health interventions and outcomes, along with creating a network of Integrative Health Centers across India, showcasing best practices, and using the portal as a continued health education tool for both patients and professionals, should be imparted gradually [25].

Research and Development

Developing practice guidelines and research protocols for integrative medicine is crucial. This includes creating guidelines for various systems of medicine and promoting research to validate the effectiveness of integrative practices [26]. Collaborative research initiatives involving institutions like AIIMS, NIHFW, and peripheral integrated health centers can drive innovation and evidence-based practices. Creating research incubation cells to foster integrated health research and innovation should be a joint effort by various research bodies.

Policy and Regulation

Implementing a regulated framework for AYUSH and developing ethical and rational use policies for integrative healthcare are necessary to tackle malpractices and ensure safe practices [27]. Effective policy implementation can support the growth of integrative medicine and enhance its credibility [28].

## Conclusions

This qualitative study highlights key themes influencing integrative medicine practices in India. While findings provide important insights into challenges and opportunities, they are exploratory in nature and should be validated through larger studies. The study contributes to understanding pathways for strengthening collaborative integrative healthcare models. The qualitative analysis of focus group discussions reveals the critical need for clarity in terminology, effective communication, trust-building, responsible integration practices, and addressing legal implications. By learning from successful integration examples and addressing identified limitations, the medical community can advance integrative medicine in clinical settings, ultimately improving patient outcomes and fostering a more holistic approach to healthcare.
